# A Small Molecule Antagonist of CX3CR1 (KAND567) Inhibited the Tumor Growth-Promoting Effect of Monocytes in Chronic Lymphocytic Leukemia (CLL)

**DOI:** 10.3390/cancers16223821

**Published:** 2024-11-13

**Authors:** Wen Zhong, Parviz Kokhaei, Tom A. Mulder, Amineh Ghaderi, Ali Moshfegh, Jeanette Lundin, Marzia Palma, Johan Schultz, Thomas Olin, Anders Österborg, Håkan Mellstedt, Mohammad Hojjat-Farsangi

**Affiliations:** 1 Department of Oncology-Pathology, BioClinicum, Karolinska University Hospital, Solna, Stockholm and Karolinska Institute, SE-17176 Stockholm, Sweden; wen.zhong.2@ki.se (W.Z.); parviz.kokhaei@ki.se (P.K.); tom.mulder@regionstockholm.se (T.A.M.); amyghd@gmail.com (A.G.); ali.moshfegh@ki.se (A.M.); jeanette.lundin@regionstockholm.se (J.L.); marzia.palma@regionstockholm.se (M.P.); anders.osterborg@regionstockholm.se (A.Ö.); hakan.mellstedt@ki.se (H.M.); 2 Department of Immunology, Arak University of Medical Sciences, Arak 3848170001, Iran; 3 Department of Hematology, Karolinska University Hospital Solna, SE-17176 Stockholm, Sweden; 4 Kancera AB, Karolinska Institutet Science Park, SE-17165 Solna, Sweden; johan.schultz@kancera.com (J.S.); thomas.olin@kancera.com (T.O.)

**Keywords:** CX3CR1, CX3CR1 antagonist, nurse-like cells, monocytes, tumor microenvironment, CLL

## Abstract

Background/Objectives: Nurse-like cells (NLCs) derived from monocytes in the tumor microenvironment support the growth of chronic lymphocytic leukemia (CLL) cells. Here, we investigated the effects of a CX3CR1 (fractalkine receptor) antagonist (KAND567) on autologous monocytes and their pro-survival effects on CLL cells in vitro. Methods: Plasma concentration of CX3CL1 was determined by ELISA and CX3CR1 expression by flow cytometry. CD19^+^ cells and autologous monocytes from patients with CLL and healthy donors were treated with KAND567 either in co-culture or alone. The apoptosis of CD19^+^ cells and monocytes was determined by Annexin V/PI staining and live-cell imaging. Results: Plasma concentration of CX3CL1 (fractalkine) was significantly higher in patients with CLL (*n* = 88) than in healthy donors (*n* = 32) (*p* < 0.0001), with higher levels in patients with active compared to non-active disease (*p* < 0.01). CX3CR1 was found on monocytes but not B cells in patients and controls. Levels of intermediate and non-classical CX3CR1^+^ monocytes were higher in patients with CLL than in controls (*p* < 0.001), particularly in those with active disease (*p* < 0.0001). Co-culture experiments revealed that autologous monocytes promoted the survival of both malignant and normal B cells and that KAND567 selectively inhibited the growth of CLL cells in a dose-dependent manner but only in the presence of autologous monocytes (*p* < 0.05). Additionally, KAND567 inhibited the transition of monocytes to NLCs in CLL (*p* < 0.05). Conclusions: Our data suggest that the CX3CR1/CX3CL1 axis is activated in CLL and may contribute to the NLC-driven growth-promoting effects of CLL cells. KAND567, which is in clinical trials in other disorders, should also be explored in CLL.

## Simple Summary

Interaction with the tumor microenvironment is of importance for the survival of tumor cells as well as response to treatment. Soluble chemokines and ligands are of significant interest. The CX3CL1/CX3CR1 axis is involved in inflammation, acting as a chemotactic cytokine. Recruiting inflammatory and immune cells to the TME. Targeting tumor growth-supporting monocytes as nurse-like cells has been suggested to be a promising therapeutic strategy. In the present study, we investigated whether autologous monocytes supported the survival of CLL cells and the potential therapeutic effects of a CX3CR1 antagonist. Our data indicated that the CX3CR1 antagonist inhibited the growth-supportive effects of CX3CR1-expressing autologous monocytes on CLL cells. No effects were observed in healthy donors. No direct effects were observed on B cells, neither from CLL patients nor from healthy donors. Further studies are warranted to explore the anti-tumor effects of the CX3CR1 antagonist in CLL as well as other malignancies.

## 1. Introduction

Chronic lymphocytic leukemia (CLL) is characterized by the accumulation of CD5^+^ B cells in lymphoid tissues [1]. The clinical manifestations of CLL vary widely, from asymptomatic non-active disease to progressive disease with potential therapeutic challenges [2]. The introduction of small molecule inhibitors of BTK and BCL-2 has dramatically improved the prognosis of CLL and increased the understanding of CLL pathobiology, but drug resistance may occur, resulting in a lack of disease control and unmet medical needs [2,3].

The interaction between tumor cells and the tumor microenvironment (TME) is important for the growth of tumor cells and treatment sensitivity [4,5]. The TME is a complex compartment, including several types of immune and inflammatory cells involved in the proliferation and survival of tumor cells as well as drug resistance [6]. In CLL, this intricate interplay is accomplished through a network of adhesion molecules, chemokines/cytokines and receptors, as well as a direct interaction between leukemic cells and stromal cells, T cells, NK cells, and specialized tumor-associated macrophages (TAM) such as nurse-like cells (NLCs) [7].

NLCs are large, oval-shaped, elongated monocytes/macrophages with an M2-like phenotype, including high surface expression of CD68, CD163, CD206, CD11b, and HLA-DR [8]. NLCs are found in secondary lymphoid organs of patients with CLL and in in vitro cultures of peripheral blood mononuclear cells (PBMCs) [9]. NLCs protect CLL cells from cell death and drug-induced apoptosis through the expression of BAFF, APRIL, CD31, plexin-B1, and brain-derived neurotrophic factor, as well as by the secretion of chemotactic chemokines such as CXCL12, CXCL13, and CCL21 [10]. Targeting the TME in addition to the tumor cells has been suggested to be a potential new treatment strategy for CLL [2].

Chemokines are a family of small cytokines ranging from 8 to 10 kDa. CX3CL1 (fractalkine) is a chemokine with a transmembrane and a soluble form, having a wide range of biological effects [11]. Soluble CX3CL1 acts as a chemotactic cytokine, while the membrane-attached variant is a binding molecule. CX3CL1 levels are increased in inflammatory disorders and cancer, with both positive and negative effects [12]. The CX3CR1 receptor (fractalkine receptor) is expressed by monocytes, dendritic cells, macrophages, T cells, and NK cells. The binding of CX3CL1 to CX3CR1 stimulates the activation of heterotrimeric G proteins associated with the receptor. CX3CR1 activates several signaling pathways, such as MAPK and AKT that are involved in tumor biology [13]. The CX3CL1/CX3CR1 axis has an anti-tumor effect by acting as a chemotactic cytokine recruiting immune cells such as NK and T cells [14], but the complex can also activate pro-tumoral responses [15]. The CX3CL1/CX3CR1 axis might be of interest in CLL and other malignancies as a therapeutic target structure [16].

The aim of the present study was to investigate the inhibitory effects of a CX3CR1 small molecule antagonist (KAND567, previously named AZD8797) on the interaction between CLL cells and the TME by co-culturing autologous monocytes with CLL cells [17]. A drug with anti-tumor effects, distinct from those directly targeting the tumor cells and complementary to such drugs, would be clinically desirable. Combining drugs that target the tumor cells and the TME may lead to potent anti-tumor responses [18].

## 2. Materials and Methods

### 2.1. Patient and Healthy Control Samples

Samples were collected from patients with CLL who were followed at the Department of Hematology, Karolinska University Hospital Solna. They had either non-active (early-stage) disease (*n* = 70) or active symptomatic disease (*n* = 29). Age-matched healthy donors (*n* = 32) were also included. The International Workshop on Chronic Lymphocytic Leukemia (iwCLL) Guidelines were used to classify non-active and active disease [19]. Ethical approval was obtained from the National Ethics Authority (https://www.government.se/government-agencies/the-swedish-ethics-review-authority-etikprovningsmyndigheten/ (accessed on 24 February 2024)). Patients and healthy donors provided written informed consent in accordance with the WMA Declaration of Helsinki before sample collection. Clinical characteristics and number of patients included in the various experiments are presented in Tables S1 and S2 and Figure S1A,B.

### 2.2. ELISA

Plasma concentration of CX3CL1 was analyzed in 29 patients with active disease, 59 patients with non-active disease, and 32 healthy donors using a human CX3CL1/Fractalkine Quantikine ELISA Kit from R&D Systems, Inc., Minneapolis, MN, USA, following the manufacturer’s instructions. A VersaMax Microplate Reader from Molecular Devices, San Jose, CA, USA, was used at 450 nm.

### 2.3. Cell Surface Expression of CX3CR1 (Flow Cytometry)

Whole blood collected from patients with CLL and healthy donors was stained with specific antibodies (Table S3) within 2 h of sampling. The blood samples were then treated with a red cell lysis solution (Beckman Coulter, Indianapolis, IN, USA) and washed with a cell staining buffer (BioLegend, San Diego, CA, USA) as previously outlined [20]. The cells were analyzed using a FACS Canto II flow cytometer (BD Bioscience, San Jose, CA, USA) and the FlowJo software (Tree Star Inc., Ashland, OR, USA, version 10.8.1).

### 2.4. Isolation of Monocytes and B Cells

PBMC were isolated by density gradient centrifugation using Ficoll-Paque Plus (Cytiva, Marlborough, MA, USA), washed, and resuspended in Phosphate-Buffered Saline (PBS) (Gibco, Life Technologies, Karlsruhe, Germany) containing 1% FBS (Gibco) and 2 mM EDTA (Ambion, Austin, TX, USA). Subsequently, the cells were incubated with anti-human CD14 antibody-conjugated Microbeads (Miltenyi Biotec, Bergisch Gladbach, Germany) and separated using LS columns (Miltenyi Biotec) to positively select CD14^+^ cells. If the CD14 negative cell fraction of CLL patients contained >80% CD19^+^ cells (flow cytometry), no further enrichment was carried out, and we considered the leukemic cell population (CD19^+^ fraction). The corresponding fraction from healthy donors always contained <80% CD19^+^ cells and was further enriched using the negative B cell isolation kit II (milltenyi Biotec) according to the manufacturer’s instruction. The purity of each cell population was analyzed by flow cytometry. The CD14^+^ fraction of CLL patients contained 94.3 ± 1.2% (mean ± SEM) CD14^+^ cells while the corresponding figures for the CD14^+^ fraction from healthy donors were 98.2 ± 0.2% CD14^+^ cells. The CD19^+^ fraction of CLL patients contained 82.7 ± 1.9% CD19^+^ cells, and the corresponding fraction of healthy donors contained 94 ± 1.7% CD19^+^ cells.

### 2.5. Annexin V/PI Apoptosis Assay and Trypan Blue Staining

Cells were washed and resuspended in RPMI 1640 (Gibco) medium containing 10% FBS, 100 μg/mL penicillin-streptomycin (Gibco), 1% L-glutamine (Gibco), and 0.2% DMSO (Sigma-Aldrich, Saint Louis, MO, USA) and referred to as the culture medium (CM). KAND567, a CX3CR1 small molecule antagonist (Kancera AB, Stockholm, Sweden) (www.kancera.com (accessed on 24 February 2024)), was dissolved in DMSO. Cells were cultured in humidified air with 5% CO2 at 37 °C. CD14^+^ and CD19^+^ fractions (10^6^ cells/mL) were either cultured separately or co-cultured (1:1) in 24-well plates (TPP, Trasadingen, Switzerland) for 120 h. Various concentrations of KAND567 were used (250, 1000, 5000 nM) in some experiments, but in most cases, 1000 nM of KAND567 was added, which corresponded to the concentration achieved in non-CLL patients who participated in ongoing clinical trials with KAND567 (Kancera AB).

CD19^+^ cell fractions cultured alone or in co-culture with CD14^+^ cells were collected every 24 h. After collection, cells were stained with Annexin V/PI (Table S3) and analyzed in flow cytometry with FlowJo software [21]. CD14^+^ monocytes cultured alone were detached at 48 and 120 h using RPMI containing 10 mM EDTA without FBS. A portion of cells was stained with a 0.4% trypan blue solution (Sigma-Aldrich) and counted in a microscope using a Bürker chamber (VWR, Stockholm, Sweden). The remaining cells were stained with Annexin V/PI.

### 2.6. CD14^+^ Monocyte Morphology Assay

Monocytes (10^5^ cells/mL) in CM containing KAND567 were seeded in a Lab-Tek II Chamber Slide (Nalge Nunc International, Rochester, NY, USA) and cultured in a humidified atmosphere at 37 °C. After 48–120 h of incubation, the CM was replaced with a solution of Nitrotetrazolium Blue Chloride (NBT) [50 mL PBS with 0.05 g NBT powder (Sigma-Aldrich), 0.85 g Bovine Serum Albumin (BSA) powder (Sigma-Aldrich), and 100 µL PMA], and then further incubated at 37 °C for 30 min. The culture chamber was removed after fixing the cells with methanol for 30 sec. The slide was washed in PBS and analyzed using a ZEISS Axio Observer microscope (20×) (Zeiss, Oberkochen, Germany) in bright field. Cell counting was performed using the Image J software (National Institute of Health, Bethesda, Maryland, USA, version 1.53k).

### 2.7. The IncuCyte Apoptosis Assay

CD14^+^ monocytes, CD19^+^ CLL cells, or healthy donor CD19^+^ cells were seeded in 96-well plates at a concentration of 10^6^ cells/mL in the CM containing Incucyte^®^ Caspase-3/7 Green Dye (1:1000) (Sartorius, Gottingen, Germany). Live-cell images (20×) were taken every 2 h (4 images per well) for 120 h using the Incucyte^®^ S3 Live-Cell analysis instrument (Sartorius). Plates were allowed to settle for 45 min before the first image capture to ensure even cell distribution. The apoptotic cell count was determined by analyzing adherent and non-adherent cells using the IncuCyte analysis software (Sartorius, version 2021A). The percentage of drug-related apoptosis at each time point was calculated as the percentage of apoptotic monocytes incubated with KAND567 minus the percentage of apoptotic monocytes in the CM alone.

### 2.8. Statistical Analysis

Statistical significance was assessed using GraphPad Prism 9 (GraphPad Software, Inc., La Jolla, CA, USA, version 9.5.1) and R software (version 4.3.1). When applicable, we employed Student’s t-test or paired t-test for repeated measurements, and used the Wilcoxon signed rank test or Wilcoxon matched-pairs signed rank test when these were not applicable. Differences between data sets were compared using a one-way ANOVA with Tukey’s multiple comparison test (Kruskal–Wallis with Dunn’s multiple comparison test for non-parametric analysis). Friedman’s 1st test was utilized to compare changes over time in patients with no missing data. For data sets with two independent categorical factors, we analyzed the influence of factors using a two-way ANOVA or Kruskal–Wallis for non-parametric comparisons. A *p*-value <0.05 was considered statistically significant.

## 3. Results

### 3.1. Plasma CX3CL1 and CX3CR1^+^ Blood Mononuclear Cells in CLL Patients

**Figure 1:**
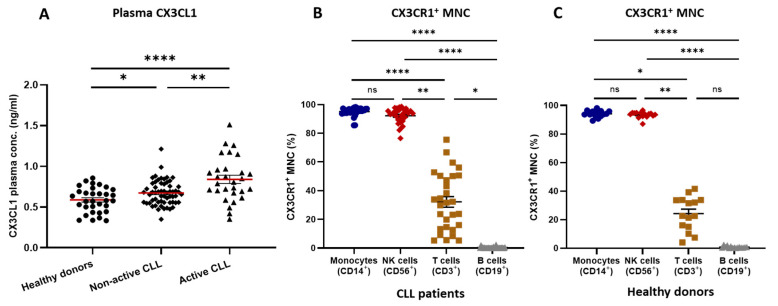
(**A**) Plasma concentration (ng/mL) of CX3CL1 of individual healthy donors (*n* = 32) as well as CLL patients with non-active (*n* = 59) and active disease (*n* = 29) as mean ± SEM. (**B**,**C**) Frequency of peripheral blood CX3CR1^+^ monocytes (CD14^+^), NK cells (CD56^+^), T cells (CD3^+^), and B cells (CD19^+^) in (**B**) CLL patients (*n* = 29) and (**C**) healthy donors (*n* = 15). Significance levels are indicated at the top. * *p* < 0.05, ** *p* < 0.01, **** *p* < 0.0001, ns: not significant, MNC: mononuclear cells.

Plasma concentrations of CX3CL1 in patients with non-active (*n* = 59) and active CLL (*n* = 29) were significantly higher than in healthy donors (*n* = 32) (*p* < 0.05 and *p* < 0.0001, respectively). Additionally, CX3CL1 concentrations in patients with active CLL were significantly higher than in non-active disease (*p* < 0.01) (Figure 1A). All monocytes in patients with CLL and healthy donors expressed CX3CR1. No expression of CX3CR1 was observed on CD19^+^ CLL cells or CD19^+^ cells of healthy donors (Figure 1B,C).

The frequency of CX3CR1 expressing NK and T cells was significantly higher than CX3CR1 expressing B cells in both CLL (Figure 1B) and healthy donors (Figure 1C).

The total number of intermediate (CD14^+^/CD16^+^) and non-classical (CD14^-^/CD16^+^) monocytes was significantly higher in patients with active CLL compared to healthy donors and to patients with non-active disease (*p* < 0.01 and *p* < 0.0001, respectively) (Figure S2). Also, the number of classical monocytes (CD14^+^/CD16^−^) was higher in patients with active than non-active CLL (*p* < 0.01) (Figure S2). No significant differences for the various monocyte subtypes were observed between patients with non-active CLL and healthy donors.

**Figure 2:**
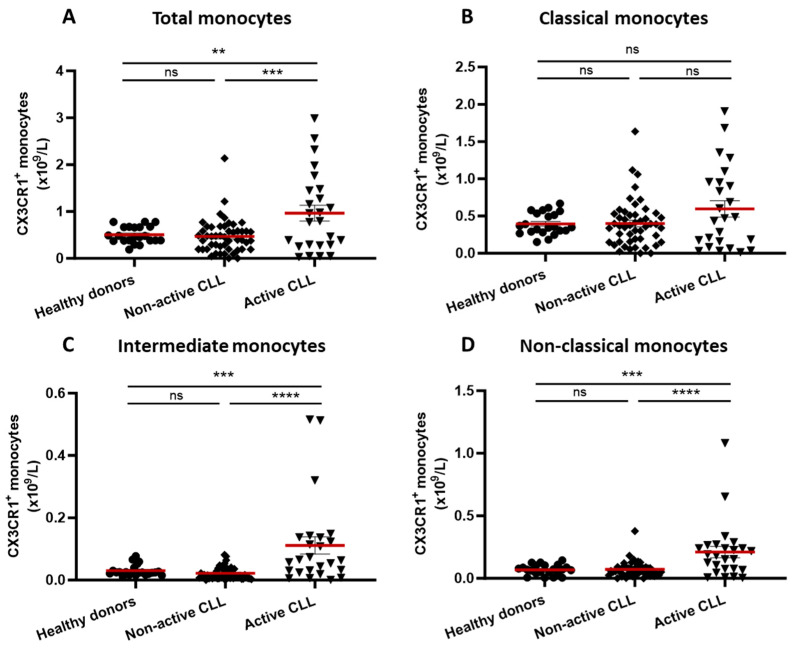
Absolute numbers (10^9^/L) (mean ± SEM) of CX3CR1^+^ monocytes in the total monocyte population (**A**) as well as in monocyte subsets (**B**–**D**) of patients with non-active (*n* = 49) and active CLL (*n* = 25) as well as healthy donors (*n* = 22). ** *p* < 0.01, *** *p* < 0.001, **** *p* < 0.0001, ns: not significant.

The total numbers of different monocyte populations expressing CX3CR1 in relation to disease activity are shown in Figure 2. There was no difference between healthy donors and patients with non-active disease, while in active disease, there was a statistically significant increase in the total numbers of intermediate and non-classical monocytes expressing CX3CR1 (*p* < 0.0001).

Non-classical monocytes in patients with CLL, as well as in healthy donors, had the highest mean fluorescence intensity (MFI) of CX3CR1 compared to intermediate and classical monocytes (Figure S3A–C). Patients with active CLL had higher CX3CR1 MFI values in classical and non-classical monocytes than those with non-active disease and healthy donors (*p* < 0.0001 and *p* < 0.01, respectively). For intermediate monocytes, no differences in MFI were seen between the groups (Figure S3E).

### 3.2. Effect on Survival of CD19^+^ CLL Cells of a CX3CR1 Antagonist

**Figure 3:**
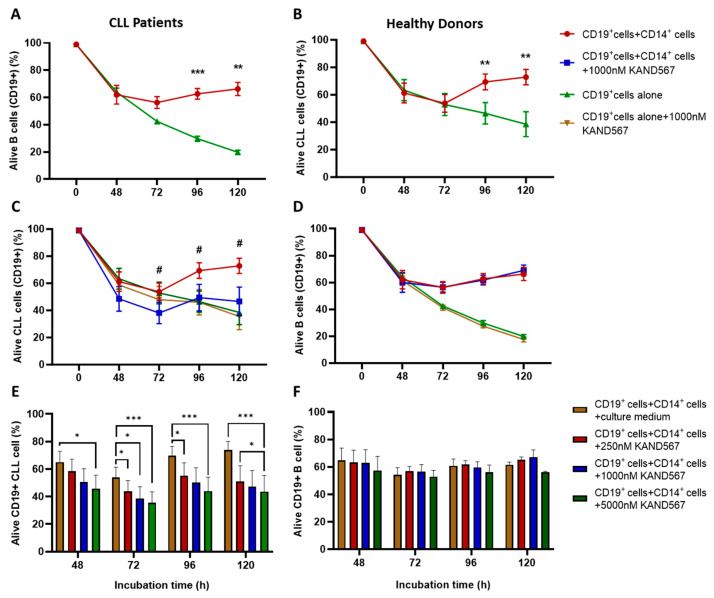
Coculture of monocytes (CD14^+^) and CD19^+^ cells from CLL patients (*n* = 15) and healthy donors (*n* = 4) in the presence of KAND567 (1000 nM). (**A**) Growth supportive effect of autologous CD14^+^ monocytes for CLL cells and (**B**) for healthy donor CD19^+^ cells. (**C**) Effect of KAND567 on alive CD19^+^ cells with and without CD14^+^ monocytes in CLL patients. (**D**) Effect of KAND567 on alive CD19^+^ cells with and without CD14^+^ monocytes in healthy donors. The frequency of alive cells was measured by (alive cells/total cells)*100. (**E**,**F**) Dose–response relationship of KAND567 on survival of CD19^+^ cells co-cultured with autologous CD14^+^ cells in CLL patients (*n* = 11) (**E**) and in healthy donors (*n* = 4) (**F**). A dose dependency was noted in CLL patients but not in healthy donors. * *p* < 0.05, ** *p* < 0.01, *** *p* < 0.001, # *p* < 0.05 comparing CD19^+^ cells and CD14^+^ cells in the presence or absence of KAND567.

After 72 h of co-culturing CD19^+^ cells with autologous CD14^+^ cells, the frequency (%) of alive CD19^+^ cells was higher in both CLL patients and healthy donors compared to CD19^+^ cells cultured alone (Figure 3A,B). However, when CLL cells and monocytes were incubated in the presence of KAND567, the frequency of alive CLL cells was significantly reduced (*p* < 0.05) (Figure 3C). KAND567 had no effect on the survival of CLL cells cultured alone (Figure 3C and Figure S4A). Monocytes from healthy donors supported the growth of normal CD19^+^ cells as well, but in contrast to CLL, KAND567 did not affect the survival of normal CD19^+^ cells when co-cultured with monocytes (Figure 3D and Figure S4B). There seemed to be a dose–response relationship for KAND567 when CD19^+^ cells from CLL patients were co-cultured with CD14^+^ cells, which was not the case for healthy donors (Figure 3E,F). The findings remained consistent when analyzing only patients for whom data for all time points were available (*n* = 6) (Figure S5), verifying the robustness of the findings. In the CD14^+^ fraction, few non-CD14^+^ cells were present (see Materials and Methods), and in the CD19^+^ fraction of CLL patients, no difference in killing in the presence or absence of KAND567 was noted, substantiating that the killing effect of KAND567 was mediated through CD14^+^ cells.

**Figure 4:**
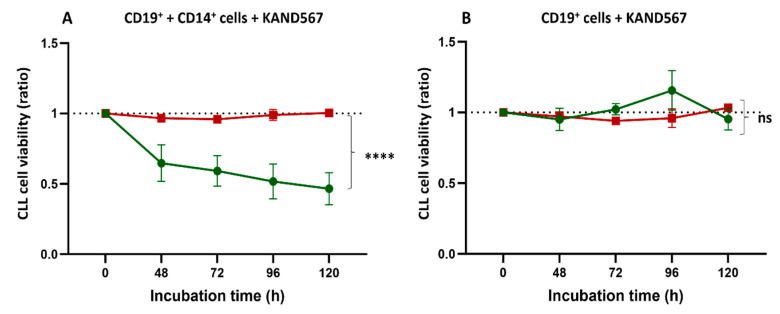
CLL cell viability (ratio) (mean ± SEM) over time in KAND567 responding # (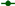) (*n* = 9) and non-responding # (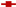) (*n* = 6) CLL patients. (**A**) CLL (CD19^+^) cell viability (%) in CD19^+^ + CD14^+^ cells + KAND567 cultures was normalized to that of CD19^+^ + CD14^+^ cells’ cultures (ratio). (**B**) CLL (CD19^+^) cell viability (%) in CD19^+^ cells + KAND567 cultures was normalized to that of CD19^+^ cell cultures alone (ratio). Statistical significance was assessed by the Kruskal–Wallis test. **** *p* < 0.0001, ns: no significance. Responding patients were defined as those with <50% alive CD19^+^ cells after 120 h of incubation, while non-responding patients were those with >50% alive CD19^+^ cells.

The effect of KAND567 varied between patients. In co-cultures of CD19^+^ cells with CD14^+^ cells and KAND567, a survival rate of CD19^+^ cells of or above 50% was observed in 6 out of 15 patients, who were categorized as non-responsive to KAND567 (Figure S6). In the remaining nine patients, <50% of leukemic cells were alive after 120 h of co-culture, categorized as responding to KAND567 (Figure S6). The difference was statistically significant (*p* < 0.0001) (Figure 4A). No difference was found when CLL cells alone were incubated with KAN567 (Figure 4B). No significant inter-individual variation was found among healthy donors (Figure S6).

### 3.3. KAND567 Inhibited Transition of Monocytes to NLCs

**Figure 5:**
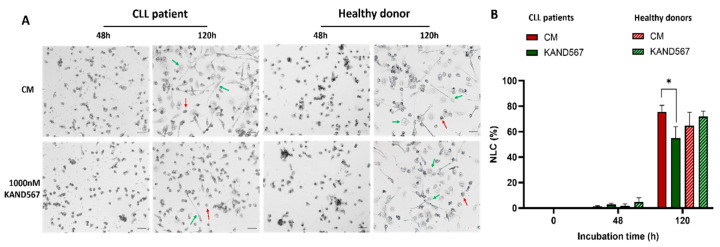
Morphological transition to NLCs of isolated monocytes (CD14^+^) from CLL patients and healthy donors incubated with KAND567. Monocytes from CLL patients (*n* = 7) and healthy donors (*n* = 5) were incubated in the absence or presence of KAND567 (1000 nM). (**A**) Microscopic photos (20×) of cells stained with NBT dye. Morphologically unchanged monocytes (red arrows) and NLC (green arrows). Scale bar = 50 μm. (**B**) Frequency of NLCs in CD14^+^ cells of CLL patients and healthy donors incubated with KAND567 (1000 nM) for 48 h and 120 h resp., * *p* < 0.05, CM: culture medium.

CD14^+^ cells from both patients with CLL and healthy donors underwent a gradual transition in vitro over a 120 h incubation period, developing into large, round, or fibroblastic-shaped adherent cells, consistent with the morphology of NLCs (Figure 5A) [10,22]. KAND567 significantly inhibited the formation of NLCs in CLL patients (*p* < 0.05) (Figure 5B), whereas no effect was observed in healthy donors (Figure 5B).

### 3.4. KAND567 Induced Cell Death/Apoptosis of CD14^+^ Cells from CLL Patients but Not from Healthy Donors

A significantly lower frequency of viable CD14^+^ cells (trypan blue staining) was observed in CLL patients after 48–120 h of incubation with KAND567 but not in CD14^+^ cells of healthy donors (Figure S7A). The CLL monocyte effect was dependent on both dose and duration of exposure to KAND567 (Figures S7B and S8).

**Figure 6:**
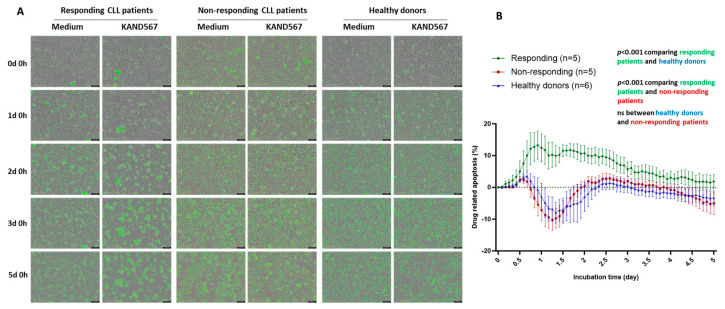
Apoptosis of CD14^+^ monocytes from CLL patients (*n* = 10) and healthy donors (*n* = 6) during a 120 h incubation period with KAND567 (1000 nM) using the Incucyte Live Imaging system. (**A**) Incucyte images of cells stained with a caspase3/7 apoptotic dye. Scale bar = 200 μm. (**B**) Drug-related (KAND567) apoptosis (%) (mean ± SEM) is identified as apoptosis in cells incubated with KAND567 apoptosis of cells in medium alone. *p*-values are shown in the figure. ns: not significant.

Finally, CD14^+^ monocytes alone from CLL patients and healthy donors were incubated with KAND567 for 120 h and analyzed for apoptosis by staining with a caspase3/7 dye using the Incucyte Live Imaging system (Figure 6A). Again, two response patterns were observed. Among the ten CLL patients tested, five showed a significant (*p* < 0.001) induction of KAND567-related apoptosis of CD14^+^ cells (responding patients) compared to healthy donors (Figure 6B). The remaining five patients did not exhibit any drug-related apoptosis (non-responding vs. responding patients) (*p* < 0.001). Healthy donor monocytes remained unaffected in this experiment.

## 4. Discussion

Understanding the micro-milieu in CLL and its interaction with the tumor clone has gained increasing interest in recent years, though therapeutic possibilities have so far been largely absent. This study explored the fractalkine axis (CX3CL1/CX3CR1) and its role in CLL. Plasma levels of the ligand (CX3CL1) and monocyte expression of the receptor (CX3CR1) were particularly high in patients with active CLL. For the first time, we also report that a small molecule antagonist of CX3CR1 (KAND567) specifically hindered the transition of monocytes into NLCs in CLL and blocked their survival-promoting effect on CLL cells in vitro, leading to tumor cell apoptosis [23].

Significant progress has been made in understanding monocyte subpopulations in health and disease. Upon release from the bone marrow, classical monocytes have the potential to transform into intermediate and non-classical monocytes. The intermediate and non-classical monocyte subsets are closely related, unlike the classical subset [24,25,26]. Studies in inflammatory diseases have shown an increase in intermediate monocytes as well as non-classical monocytes [27,28]. Monocytes are also important in the pathogenesis of various cancers [29,30].

A recent study revealed a significantly elevated number of circulating intermediate and non-classical monocytes in patients with active CLL [31]. Compared to classical monocytes, these activated subtypes could secrete high levels of TNF-α and IL-1β, cytokines that may play a role in the progression of CLL [31]. Another study reported that a decrease in the number of non-classical monocytes may be linked to CLL progression [32].

The survival of monocytes may partly rely on the CX3CR1/CX3CL1 axis, which stimulates the expression of anti-apoptotic genes such as *BCL-2* and *BCL-xL* [33,34]. Our data indicated that the density of CX3CR1 was more pronounced in non-classical monocytes and increased with the CX3CL1 plasma concentration and CLL disease activity. These findings align with the observation of elevated blood levels of TNF-α in CLL [32]. TNF-α, secreted by non-classical monocytes, enhanced the expression of CX3CL1 at both mRNA and protein levels [31,35]. In previous studies, it has been observed that while NLCs from CLL patients and healthy donors share a similar morphology, there are differences in the expression of phenotypic molecules and production of cytokines/chemokines [8,10]. It has been suggested that disrupting the formation of NLCs in CLL could be a novel therapeutic concept [8,10,36,37] as monocytes/NLCs have been found to support the growth of CLL cells and contribute to treatment resistance [38,39]. Fully differentiated NLCs have been observed to mature from PBMCs or CD14^+^ monocytes of CLL patients within 14 days of in vitro culture [8,10,36]. Trametinib, an inhibitor of MEK in the MAPK kinase pathway, did not have a direct cytotoxic effect on CLL cells but inhibited the maturation of monocytes into NLCs. It also induced apoptosis/necrosis of monocytes and increased the survival of mice in the Eµ-TCL1 CLL mouse model [36]. Our results align with these findings; as a CX3CR1 antagonist, KAND567, inhibited the transition of CLL-related monocytes to NLCs and suppressed their CLL-supporting effect, leading to CLL cell apoptosis.

Monocytes may have been activated within the CLL microenvironment by chemokines/cytokines secreted by the leukemic cells, as well as via cell-to-cell contact [40,41]. This is consistent with the findings of Boissard et al. [10], which suggest that the transition of CD14^+^ monocytes to NLCs in CLL may involve abnormal modifications, while CD14^+^ monocytes in healthy donors undergo natural maturation to NLCs [10].

The fractalkine (CX3CR1/CX3CL1) axis plays an important role in hyper-inflammation and has been identified as a potential target for treating inflammatory diseases as well as solid cancers [42]. Previous research has shown that KAND567 non-competitively displaced CX3CL1 from CX3CR1, affecting the adhesion and chemotaxis of monocytes [17,35].

The interaction between CX3CL1 and CX3CR1 is crucial for the survival of normal monocytes [35,36,43]. For the sustenance of CLL-related monocytes, CX3CR1 may be necessary but not CX3CL1 [43]. In CLL, there is an upregulation in monocytes of genes related to the Raf/ERK signaling pathway [44]. Activation of the Raf/ERK signaling pathway is essential for the downstream signaling of CX3CR1 and monocyte survival [35,44]. This may explain why CLL-related monocytes and healthy donor monocytes responded differently to KAND567, as well as why blocking the MAPK/ERK pathway is also involved in the downstream signaling of CX3CL1/CX3CR1 and monocyte survival [35,36].

KAND567 induced apoptosis of CLL-related monocytes in the absence of exogenous CX3CL1. Human monocytes seemed to be unable to produce CX3CL1 when cultured alone in vitro, and the addition of exogenous CX3CL1 had a substantial anti-apoptotic effect involving a reduction in oxidative stress [43]. The findings may indicate that the effect of KAND567 is specific for monocytes in patients with CLL compared to healthy donor monocytes and might not be related to the surface expression of CX3CR1 [43]. KAND567 is an allosteric modulator of CX3CR1 and is hypothesized to bind intracellularly near the C-terminus of CX3CR1 [17].

In approximately half of our tested patients, KAND567 inhibited the CLL growth-supportive effect of monocytes. Additionally, monocytes underwent apoptosis after exposure to KAND567 in many but not all patients. A previous study [22] demonstrated heterogeneity of monocytes in patients with CLL, noting the absence of NLC development in vitro in about half of the patients. Furthermore, NLCs generated in vitro from patients exhibited varying proportions of mature CD163^+^ NLCs, which correlated with the prognosis of both CLL and aggressive lymphomas. This was associated with the secretion of CCL21 by CD163^+^ NLC [7,45,46]. Other studies have also indicated differences in NLC maturation in CLL [39,47]. The varying effects of KAND567 may be attributed to NLC differentiation.

There are several limitations in the study. First, the number of cell co-culture experiments was limited. Second, cells could not be analyzed at all time points and in all assays simultaneously due to shortage of monocytes. We also had to limit the functional analyses to the total CD14^+^ monocyte population rather than co-culturing enriched CD14^+^ subtypes with CLL cells in refined analyses including different CX3CR1 expression levels [48]. Due to the low number of isolated monocytes available from the blood of patients with CLL, resulting in also low numbers of NLCs, we were unable to analyze intracellular pathways and mechanisms for the KAND567-induced blocking of the differentiation of monocytes to NLCs in CLL. Further studies are planned to address these questions in in vitro and CLL mouse models, including biological differences between responding and non-responding patients.

Further studies are necessary to fully grasp the impact of KAND567 on monocyte functions in CLL and related disorders. Importantly, KAND567 has already underwent toxicity/tolerability studies in healthy donors. Phase 2 trials are ongoing based on MTD in patients with ovarian carcinoma and acute myocardial infarction (Kancera AB, data on file). Clinical trials of KAND567 in patients with double-refractory CLL are warranted.

## 5. Conclusions

The tumor microenvironment (TME) plays an important role in supporting the survival and progression of tumor cells. The fractalkine axis (CX3CL1/CX3CR1) is a key factor in the interaction between tumor cells and growth-supporting cells in the TME. The present study has demonstrated that a CX3CR1 antagonist (KAND567) induced apoptosis of monocytes in CLL and inhibited monocyte maturation to NLCs, “nurse-like cells” The effect of KAND567 was specific for CLL as compared to healthy donors. Additional clinical trials are warranted in CLL and related disorders to analyze the in vivo mechanisms of action of KAND567.

## Data Availability

Data are contained within the article and Supplementary Materials.

## Supplementary Materials

The following supporting information can be downloaded at: https://www.mdpi.com/article/10.3390/cancers16223821/s1. Figure S1. (A) Age (median) of CLL patients with non-active (*n* = 70) and active CLL (*n* = 29) as well as healthy donors (*n* = 32). (B) Flow chart of the study showing the numbers of patients and healthy donors enrolled in the different assays. Figure S2. (A) (A) Absolute numbers (109/L) (mean ± SEM) of total monocytes and subtypes in patients with non-active (*n* = 49) and active CLL (*n* = 25) as well as healthy donors (*n* = 22). (B) Percentage (mean ± SEM) of monocyte subtypes in patients with non-active (*n* = 60) and active CLL (*n* = 25) as well as healthy donors (*n* = 30). Significance levels are indicated at the top. * *p* < 0.05, ** *p* < 0.01, *** *p* < 0.001, **** *p* < 0.0001, ns: not significant. Figure S3. CX3CR1 MFI value on monocyte subtypes in patients with non-active (*n* = 60) and active CLL (*n* = 25), as well as in healthy donors (*n* = 30). Significance levels are indicated at the top. ** *p* < 0.01, *** *p* < 0.001, **** *p* < 0.0001, ns: not significant. Figure S4. Apoptosis (mean ± SEM) in CD19^+^ cells from (A) CLL patients (*n* = 12) and (B) healthy donors (*n* = 6) incubated with KAND567 (1000 nM) compared to culture medium alone. Apoptosis in CD19^+^ cells was detected by Caspase3/7 staining. Data were collected for 5 days by Incucyte Live Cell Imaging. Figure S5. Frequency of alive B cells (mean ± SEM) over time in the same CLL patients (*n* = 6) and healthy donors (*n* = 4). (A) CD19^+^ cells cultured alone. (B) CD19^+^ cells with autologous CD14^+^ monocytes. (C) CD19^+^ cells cocultured with autologous CD14^+^ monocytes in the presence of KAND567 (1000 nM). (D) CD19^+^ cells alone incubated with KAND567. * *p* < 0.05, ** *p* < 0.01, *** *p* < 0.001, **** *p* < 0.0001, ns: not significant. Figure S6. Individual survival curves for (A) leukemic CD19^+^ cells from CLL patients (*n* = 15) and (B) CD19^+^ cells from healthy donors (*n* = 4). CD19^+^ cells were cultured alone, with autologous monocytes, with autologous monocytes plus KAND567 (1000 nM), and alone with KAND567, respectively. Non-responding patients () (*n* = 6) were defined as those with >50% alive CD19^+^ cells in co-cultures with KAND567 (1000 nM) for 5 days. Responding patients ( ) (*n* = 9) were those with <50% alive CD19^+^ cells in 5 days of culture. Figure S7. Cell death (trypan blue staining) (mean ± SEM) in isolated CLL CD14^+^ monocytes incubated with KAND567 for 48–120 h. (A) Freq. (%) (mean ± SEM) of alive CD14^+^ monocytes from CLL patients (*n* = 13) and healthy donors (*n* = 7). (B) Dose and time dependency of alive CD14^+^ monocytes from CLL patients (*n* = 13) incubated with KAND567. * *p* < 0.05, ** *p* < 0.01, *** *p* < 0.001, **** *p* < 0.0001, CM: culture medium. Figure S8. CLL cell viability (%) (mean ± SEM) over time in responding# (*n* = 9) and non-responding# (*n* = 6) CLL patients. Statistical significance at each time point was analyzed by an unpaired t-test. * *p* < 0.05, ** *p* < 0.01, *** *p* < 0.001, **** *p* < 0.0001. ^#^ Responding patients were defined as those with <50% alive CD19^+^ cells after 120 h of incubation, while non-responding patients were those with >50% alive CD19^+^ cells. Table S1. Clinical characteristics of the patients included in cell co-culture experiments (Figure 3 and Figure 4). Table S2. Clinical characteristics of the patients included in the monocyte apoptosis assay (Figure 6). Table S3. Antibodies used for flow cytometry.
