# Supplementary material for: A Small Molecule Antagonist of CX3CR1 (KAND567) Inhibited the Tumor Growth-Promoting Effect of Monocytes in Chronic Lymphocytic Leukemia (CLL)
Source: Cancers (Basel). 2024 Nov 13;16(22):3821. doi: 10.3390/cancers16223821 (PMC11592364; doi:10.3390/cancers16223821)
Supplement: Supplementary file 1 [file cancers-16-03821-s001.zip › cancers-3244349-supplementary.pdf]

A small molecule antagonist of CX3CR1 (KAND567) inhibited the tumor growth-promoting effect of monocytes in chronic lymphocytic leukemia (CLL)

Wen Zhong [1], Parviz Kokhaei [1, 2], Tom Mulder [1,3], Amineh Ghaderi [1], Ali Moshfegh [1], Jeanette Lundin [1,3], Marzia Palma [1,3], Johan Schultz [4], Thomas Olin [4], Anders Österborg [1,3], Håkan Mellstedt [1] and Mohammad Hojjat Farsangi [1, *]

[1] Department of Oncology-Pathology, BioClinicum, Karolinska University Hospital Solna and Karolinska Institutet, Stockholm, Sweden; wen.zhong.2@ki.se; parviz.kokhaei@ki.se; tom.mulder@regionstockholm.se; amyghd@gmail.com; Ali.Moshfegh@ki.se; jeanette.lundin@regionstockholm.se; marzia.palma@regionstockholm.se; anders.osterborg@regionstockholm.se; hakan.mellstedt@ki.se; mohammad.hojat-farsangi@ki.se
[2] Department of Immunology, Arak University of Medical Sciences, Arak, Iran,
[3] Department of Hematology, Karolinska University Hospital Solna, Stockholm, Sweden
[4] Kancera AB, Karolinska Institutet Science Park, Stockholm, Sweden; johan.schultz@kancera.com; thomas.olin@kancera.com

* Correspondence: mohammad.hojat-farsangi@ki.se; Tel.: +46 735234706

Supplementary figures and tables:

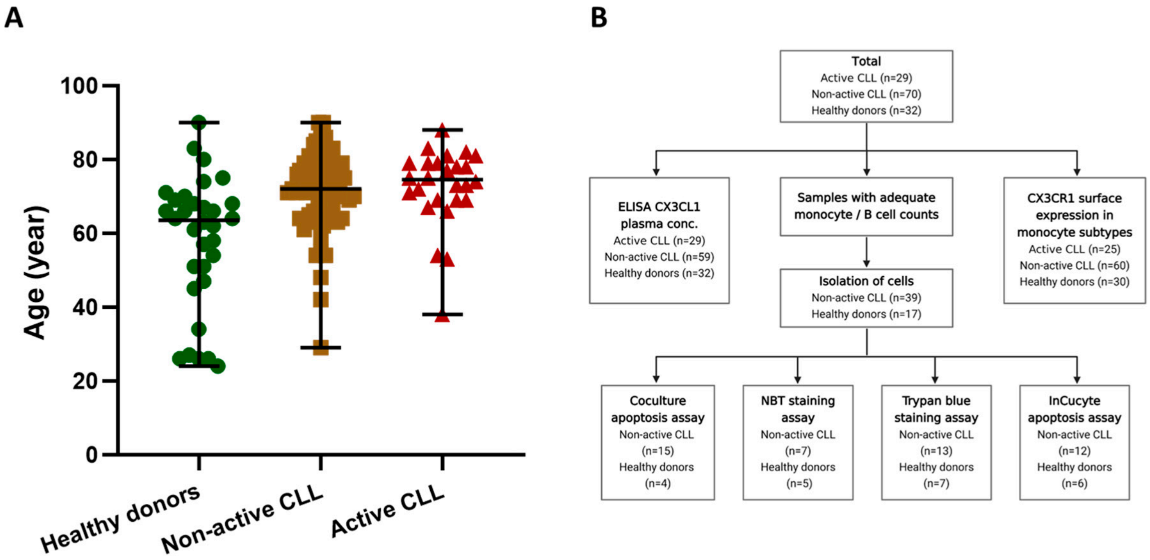

Figure S1. (A) Age (median) of CLL patients with non-active ($n = 70$) and active CLL ($n = 29$) as well as healthy donors ($n = 32$). (B) Flow chart of the study showing the numbers of patients and healthy donors enrolled to the different assays.

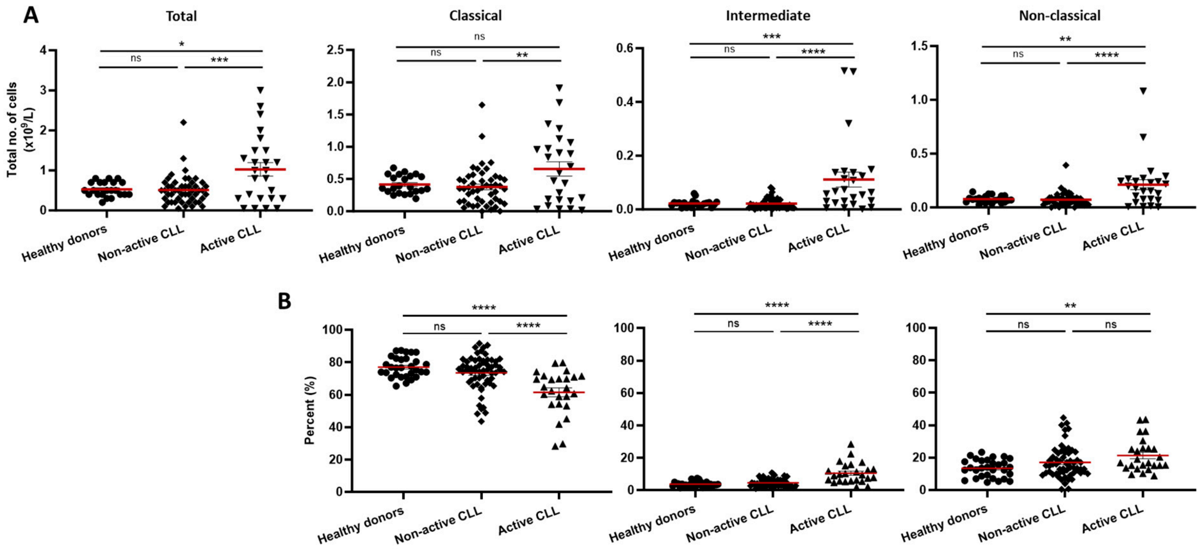

Figure S2. (A) Absolute numbers (10^9/L) (mean ± SEM) of total monocytes and subtypes in patients with non-active (n = 49) and active CLL (n= 25) as well as healthy donors (n = 22). (B) Percentage (mean ± SEM) of monocytes subtypes in patients with non-active (n = 60) and active CLL (n = 25) as well as healthy donors (n = 30). Significance levels are indicated at the top. * $p < 0.05$, ** $p < 0.01$, *** $p < 0.001$, **** $p < 0.0001$, ns: not significant.

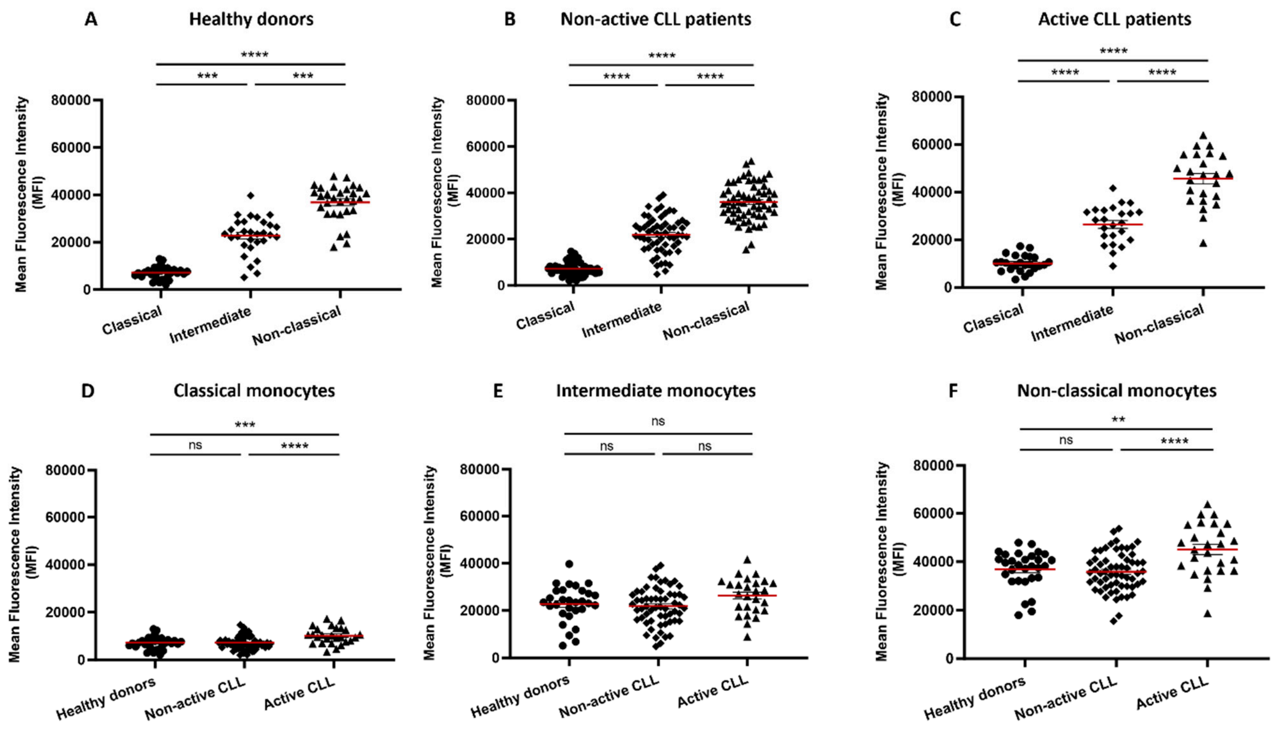

Figure S3. CX3CR1 MFI value on monocyte subtypes in patients with non-active (n = 60) and active CLL (n = 25) as well as in healthy donors (n = 30). Significance levels are indicated at the top. ** $p < 0.01$, *** $p < 0.001$, **** $p < 0.0001$, ns: not significant.

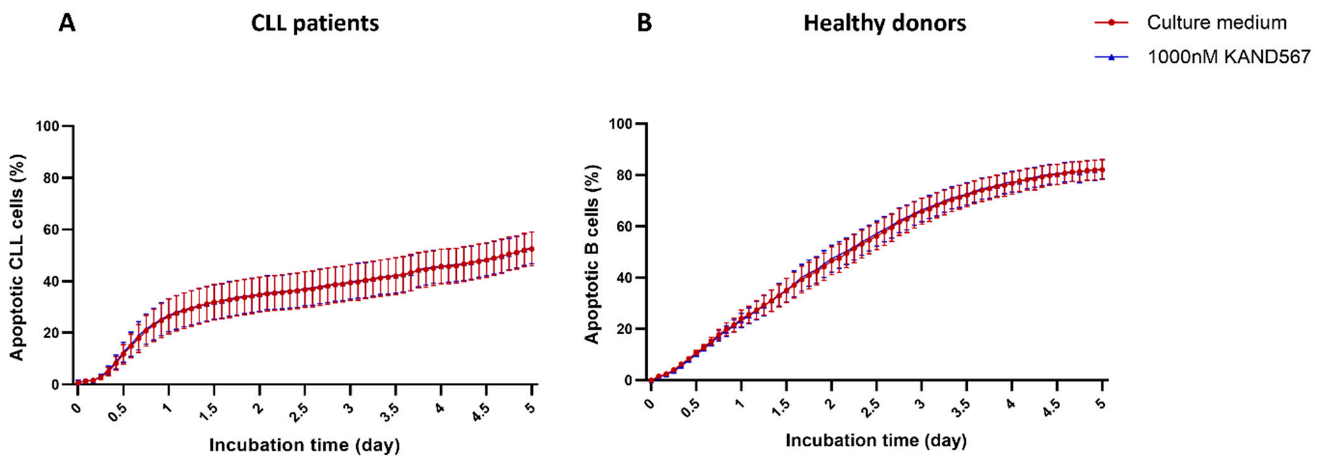

Figure S4. Apoptosis (mean ± SEM) in CD19+ cells from (A) CLL patients (*n* = 12) and (B) healthy donors (*n* = 6) incubated with KAND567 (1000 nM) compared to culture medium alone. Apoptosis in CD19+ cells was detected by Caspase3/7 staining. Data was collected for 5 days by Incucyte Live Cell Imaging.

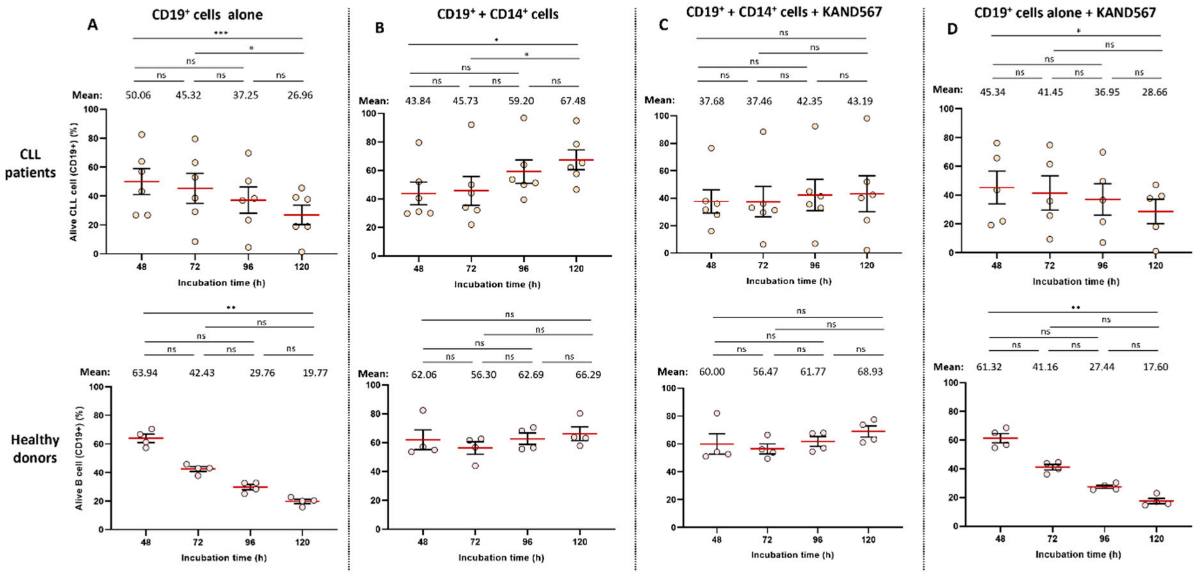

Figure S5. Frequency of alive B cells (mean ± SEM) over time in the same CLL patients (*n* = 6) and healthy donors (*n* = 4). (A) CD19+ cells cultured alone. (B) CD19+ cells with autologous CD14+ monocytes. (C) CD19+ cells cocultured with autologous CD14+ monocytes in the presence of KAND567 (1000 nM). (D) CD19+ cells alone incubated with KAND567. * p<0.05, ** p<0.01, *** p<0.001, **** p<0.0001, ns: not significant.

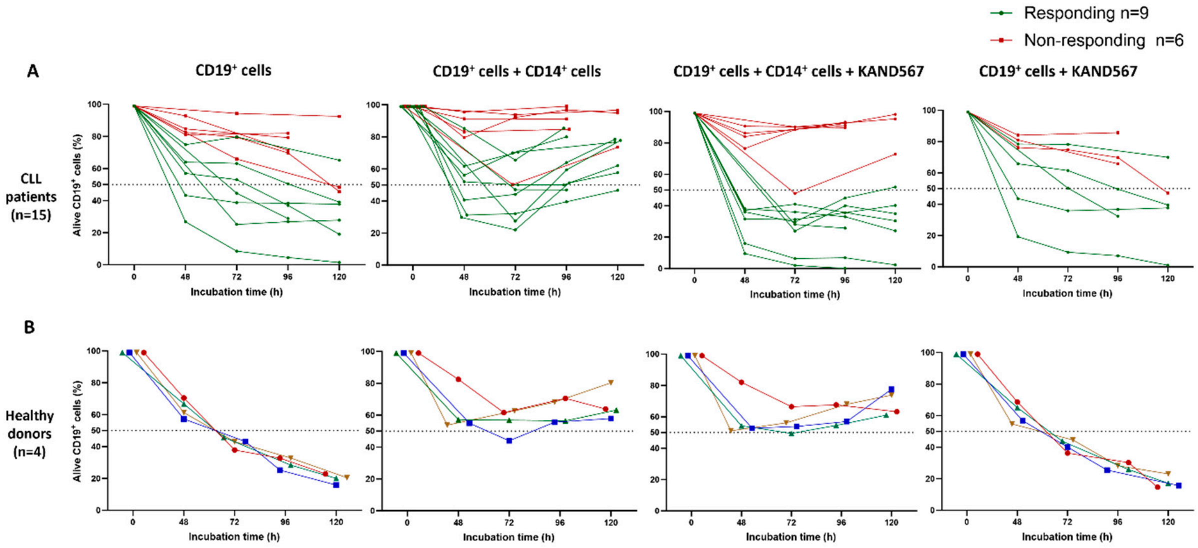

Figure S6. Individual survival curves for (A) leukemic CD19+ cells from CLL patients (*n* = 15) and (B) CD19+ cells from healthy donors (*n* = 4). CD19+ cells were cultured alone, with autologous monocytes, with autologous monocytes plus KAND567 (1000 nM), and alone with KAND567, respectively. Non-responding patients (—■—) (*n* = 6) were defined as those with >50% alive CD19+ cells in co-cultures with KAND567 (1000 nM) for 5 days. Responding patients (—●—) (*n* = 9) were those with < 50% alive CD19+ cells in 5 days of culture.

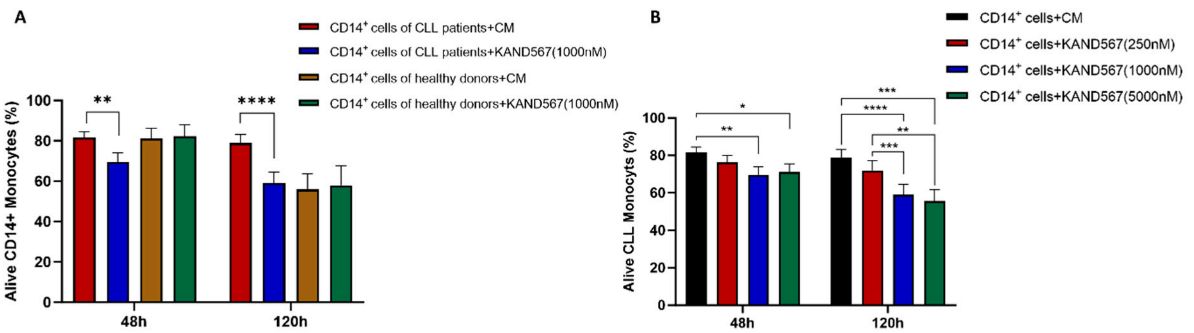

Figure S7. Cell death (trypan blue staining) (mean ± SEM) in isolated CLL $CD14^+$ monocytes incubated with KAND567 for 48-120 h. (A) Freq. (%) (mean ± SEM) of alive $CD14^+$ monocytes from CLL patients ($n = 13$) and healthy donors ($n = 7$). (B) Dose and time dependency of alive $CD14^+$ monocytes from CLL patients ($n = 13$) incubated with KAND567. * $p < 0.05$, ** $P < 0.01$, *** $p < 0.001$, **** $p < 0.0001$, CM: culture medium.

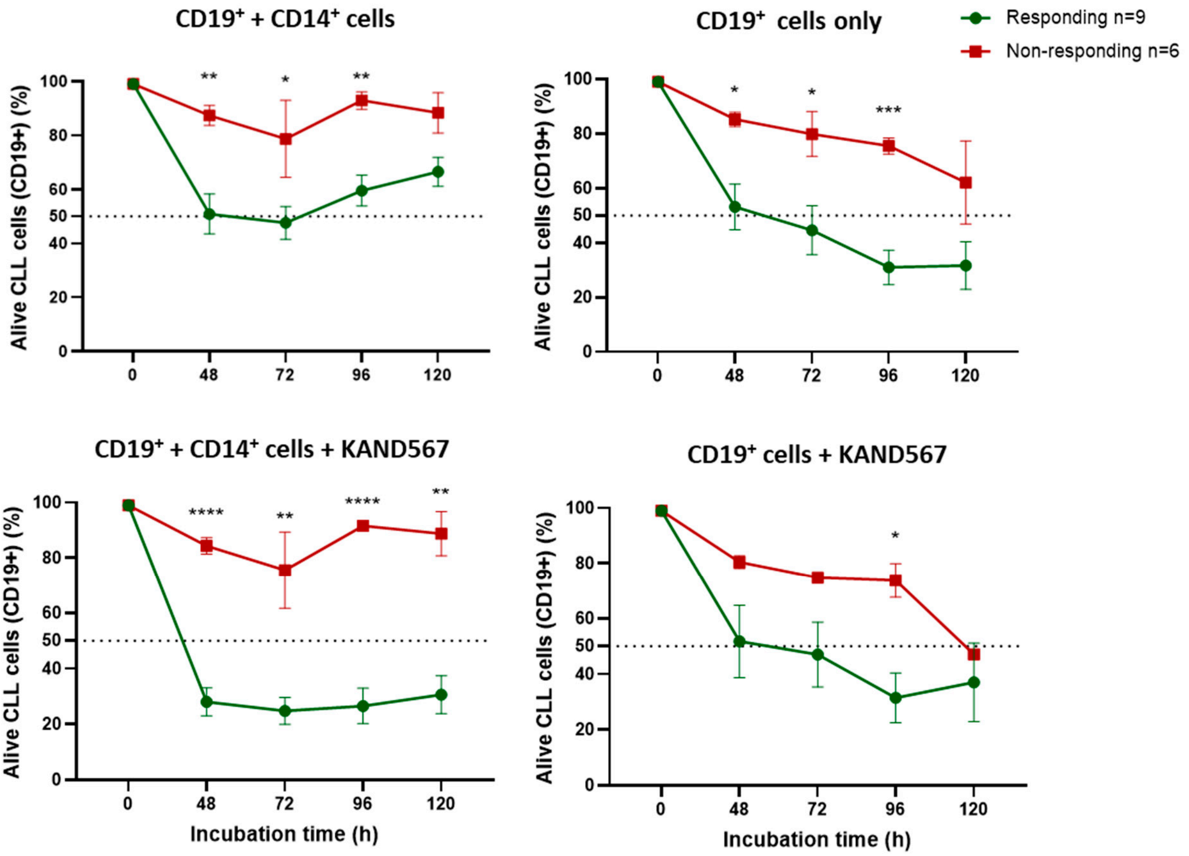

Figure S8. CLL cell viability (%) (mean±SEM) over time in responding# (n = 9) and non-responding# (n = 6) CLL patients. Statistical significance at each time point was analysed by unpaired t test. * $p < 0.05$, ** $p < 0.01$, *** $p < 0.001$, **** $p < 0.0001$. # Responding patients were defined as those with < 50% alive CD19+ cells after 120 h of incubation, while non-responding patients were those with > 50% alive CD19+ cells.

Table S1. Clinical characteristics of the patients included in cell co-culture experiments (Figs.3-4).

Patient No.	Age (Y) – Gender (Female / Male)	Disease activity at sampling (Progressive / Non-progressive)	Rai stage	WBC count ($x10^9/L$)	Lymphocyte count ($x10^9/L$)	Monocyte count ($x10^9/L$)	Platelet count ($x10^9/L$)	Previous Treatment (time since last therapy)	IGHV Mutation	TP53 Mutation	β2M (mg/L)
1	58 --F	Active	0	46.4	41.9	0.9	207	No	Unmutated	No	2.2
2	54 --F	Non-active	1	25.3	16.8	0.8	408	No	ND	ND	ND
3	72 --M	Non-active	1	31.4	27.9	<0.1	128	No	ND	ND	ND
4	62 --F	Active	0	22.9	22.9	0.6	171	Yes (4.1 years) *	ND	No	ND
5	54 --M	Non-active	2	25.1	25.1	ND	207	No	Mutated	No	5.8
6	65 --F	Non-active	0	24.1	21.3	0.4	179	No	ND	ND	ND
7	77 --F	Non-active	0	14.4	6.8	0.8	305	No	ND	ND	1.7
8	78 --F	Non-active	1	31.4	25.4	ND	209	No	ND	ND	ND
9	75 --F	Non-active	1	16.4	12.2	0.6	164	No	ND	ND	ND
10	84 --M	Non-active	1	11.3	4.2	0.9	213	No	ND	ND	2.4
11	83 --M	Non-active	0	22.3	18.1	0.6	110	No	ND	ND	3.6
12	76 --M	Active	0	38.6	31.5	1	173	No	ND	ND	ND
13	79 --M	Non-active	1	11.3	4.6	0.7	252	Yes (2.6 years) #	ND	ND	ND
14	70 --M	Non-active	0	19.3	14.4	0.1	248	No	ND	ND	ND
15	71 --M	Non-active	1	23.2	19.8	0.5	213	No	ND	ND	ND

ND = Not done; WBC = White Blood Cell

* Last treatment was performed in Nov 2017 with FCR regimen

Last treatment was performed in Oct 2019 with BR regimen

Table S2. Clinical characteristics of the patients included in monocyte apoptosis assay (Fig.6).

Patient No.	Age (Y) – Gender (Female / Male)	Disease activity at sampling (Progressive / Non-progressive)	Rai stage	WBC count ($x10^9/L$)	Lymphocyte count ($x10^9/L$)	Monocyte count ($x10^9/L$)	Platelet count ($x10^9/L$)	Previous Treatment (time since last therapy)	IGHV Mutation	TP53 Mutation	β2M (mg/L)
34	64 --M	Non-active	1	32.8	26.4	0.6	205	No	Mutated	ND	3
35	78 --M	Non-active	0	27.9	23.5	0.6	208	No	Mutated	No	2.2
36	64 --F	Non-active	1	36.8	29.8	0.6	157	No	Unmutated	No	2.4
37	85 --M	Non-active	0	15.6	9	0.7	238	No	ND	ND	4.6
38	64 --F	Non-active	1	22.8	19.9	0.1	170	No	ND	ND	ND
41	72 --F	Non-active	0	18.9	15.9	0.3	186	No	ND	ND	ND
42	29 --M	Non-active	1	13	8.8	0.5	244	No	Unmutated	No	1.8
45	72 --M	Non-active	0	9.5	5.7	0.4	146	No	ND	ND	ND
46	79 --F	Non-active	1	15.5	9.2	0.4	218	No	Unmutated	No	ND
47	61 --M	Non-active	0	21.1	14.2	0.6	198	No	ND	ND	1.7

ND = Not done; WBC = White Blood Cell

Table S3. Antibodies used for flow cytometry.

Panel 1

Target	Fluorochrome	Clone	Company	Cat. No.
**CD3**	FITC	UCHT1	BioLegend, San Diego, CA, USA	300406
**CD19**	FITC	HIB19	BioLegend, San Diego, CA, USA	302206
**CD56**	FITC	HCD56	BioLegend, San Diego, CA, USA	318304
**CX3CR1**	PE	2A9-1	BioLegend, San Diego, CA, USA	341604
**CD14**	PerCp	MφP9	BD Bioscience, San Jose, CA, USA	340660
**CD16**	APC	B73.1	BioLegend, San Diego, CA, USA	360706
**CD45**	Alexa Fluor 700	HI30	BioLegend, San Diego, CA, USA	304024

Panel 2

Target	Fluorochrome	Clone	Company	Cat. No.
**CD45**	Alexa Fluor 700	HI30	BioLegend, San Diego, CA, USA	304024
**CD19**	PE/Cyanine 7	HIB19	BioLegend, San Diego, CA, USA	302216
**Annexin V**	FITC	-	BioLegend, San Diego, CA, USA	640945
**PI**	-	-	BD Bioscience, San Jose, CA, USA	556463

Panel 3

Target	Fluorochrome	Clone	Company	Cat. No.
**CD45**	V450	HI30	BD Bioscience, San Jose, CA, USA	560367
**CD14**	PerCp	MφP9	BD Bioscience, San Jose, CA, USA	340660
**CD16**	APC	B73.1	BioLegend, San Diego, CA, USA	360706
**Annexin V**	FITC	-	BioLegend, San Diego, CA, USA	640945
**PI**	-	-	BD Bioscience, San Jose, CA, USA	556463
